# *LMO1* gene polymorphisms contribute to decreased neuroblastoma susceptibility in a Southern Chinese population

**DOI:** 10.18632/oncotarget.8178

**Published:** 2016-03-18

**Authors:** Jing He, Wei Zhong, Jixiao Zeng, Jinhong Zhu, Ruizhong Zhang, Fenghua Wang, Tianyou Yang, Yan Zou, Huimin Xia

**Affiliations:** ^1^ Department of Pediatric Surgery, Guangzhou Institute of Pediatrics, Guangzhou Women and Children's Medical Center, Guangzhou Medical University, Guangzhou 510623, Guangdong, China; ^2^ Sun Yat-Sen University Cancer Center, State Key Laboratory of Oncology in South China, Department of Experimental Research, Collaborative Innovation Center for Cancer Medicine, Guangzhou 510060, Guangdong, China; ^3^ Molecular Epidemiology Laboratory and Department of Laboratory Medicine, Harbin Medical University Cancer Hospital, Harbin 150040, Heilongjiang, China

**Keywords:** neuroblastoma, LMO1, polymorphism, genetic susceptibility

## Abstract

Neuroblastoma is one of the most commonly diagnosed extracranial solid tumors in infancy; however, the etiology of neuroblastoma remains largely unknown. Previous genome-wide association study (GWAS) indicated that several common genetic variations (rs110419 A > G, rs4758051 G > A, rs10840002 A > G and rs204938 A > G) in the *LIM domain only 1 (LMO1)* gene were associated with neuroblastoma susceptibility. The aim of this study was to evaluate the correlation between the four GWAS-identified *LMO1* gene polymorphisms and neuroblastoma risk in a Southern Chinese population. We genotyped the four polymorphisms in 256 neuroblastoma cases and 531 controls. Odds ratios (ORs) and 95% confidence intervals (CIs) were used to evaluate the strength of the associations. False-positive report probability was calculated for all significant findings. We found that the rs110419 A > G polymorphism was associated with a significantly decreased neuroblastoma risk (AG vs. AA: adjusted OR = 0.65, 95% CI = 0.47–0.91; GG vs. AA: adjusted OR = 0.58, 95% CI = 0.36–0.91; AG/GG vs. AA: adjusted OR = 0.63, 95% CI = 0.46–0.86), and the protective effect was more predominant in children of age > 18 months, males, subgroups with tumor in adrenal gland and mediastinum, and patients in clinical stages III/IV. These results suggested that *LMO1* gene rs110419 A > G polymorphism may contribute to protection against neuroblastoma. Our findings call for further validation studies with larger sample size.

## INTRODUCTION

Neuroblastoma is an embryonic cancer that arises from primordial cells during fetal or early childhood development [1]. It is also the most commonly diagnosed extracranial solid tumor in childhood, accounting for more than 7% of malignancies in patients younger than 15 years [1, 2]. In the United States, the incidence rate of neuroblastoma is about 1 in 7000 live newborns [3], while the rate is roughly 7.7 per million in China [4]. Approximately, 1% of the neuroblastoma patients have a family history and they are generally diagnosed at a much earlier age and more prone to develop multifocal primary tumors [5, 6]. Cure rates for high-risk neuroblastoma patient remains less than 40% [7, 8], and the 5-year survival rate for neuroblastoma patients is around 70%. Nearly 6% of patients died from recurrence or second tumor after 5 years of diagnosis [9]. Neuroblastoma has devastating impacts on affected family and is also a great challenge for public health [10].

In order to identify risk factors for neuroblastoma, a multitude of epidemiological studies have been performed to investigate some putative causative agents and their biological effects in different populations [11]. Unfortunately, so far, epidemiological studies haven't identified any common environmental exposure that can definitely influence neuroblastoma susceptibility [12, 13]. On the other hand, molecular epidemiological studies focus on the use of biomarkers in epidemiological research, which are typically indicators of exposure, effect, or susceptibility. Accumulating evidence from molecular epidemiological studies suggests that genetic factors may play a critical role in the development of neuroblastoma [14–18].

Genome-wide association study (GWAS) has served as a powerful tool in the identification of inherited genetic variations that are associated with complex human diseases including cancer [19]. Previous GWASs have discovered several inherited common variants in some chromosomal regions that are significantly associated with the risk of neuroblastoma, such as *LINC00340* (also known as *FLJ22536* or *CASC15*) at 6p22 [1], *BARD1* at 2q35 [20], and *LIM domain only 1* (*LMO1*) at 11p15 [21]. In a GWAS limited to the European descent, Wang et al. [21] recruited a total of 1627 neuroblastoma cases and 3254 controls in the discovery stage to screen neuroblastoma susceptibility loci. Next, the four most significant variants identified in the first phase were further validated in 190 neuroblastoma cases and 1507 controls from United States, 253 cases and 845 controls from United Kingdom, as well as 181 cases and 491 controls from Italy. They found that four single nucleotide polymorphisms (SNPs) in the *LMO1* gene (rs110419 A > G, rs4758051 G > A, rs10840002 A > G and rs204938 A > G) were associated with neuroblastoma susceptibility. Since then, the association between the four *LMO1* SNPs and neuroblastoma have been validated in African-Americans [22], Italians [23], and a Northern Chinese population [24]. There may exist significant differences in genetic background between Europeans and Chinese subjects, and the differences may even exist among the different regions of China, which may modify the association between SNPs and diseases including neuroblastoma. With these in mind, in this hospital-based case-control study, we aimed to determine the relationship between *LMO1* gene polymorphisms and neuroblastoma susceptibility in a Southern Chinese population with 256 cases and 531 controls.

## RESULTS

### Population characteristics

As shown in Table 1, a total of 256 neuroblastoma cases and 531 age-, gender- and ethnicity-matched controls were included in the current study. Briefly, no significant differences were observed in age (30.87 ± 26.45 vs. 29.73 ± 24.86, *P* = 0.239) and gender (*P* = 0.333) between neuroblastoma cases and healthy controls. Of the neuroblastoma cases, 54 (21.09%) were diagnosed with clinical stage I, 65 (25.39%) with clinical stage II, 44 (17.19%) with clinical stage III, 77 (30.08) with clinical stage IV, and 9 (3.52%) with clinical stage 4s disease, according to the INSS criteria [25]. Moreover, 46 (17.97%) neuroblastomas were developed in adrenal gland, 87 (33.98%) in retroperitoneal region, and 90 (35.16%) in the mediastinum.

**Table 1:** Frequency distribution of selected characteristics in neuroblastoma patients and controls

Variables	Cases (*n* = 256)		Controls (*n* = 531)		*P*^a^
	No.	%	No.	%	
Age range, month	0–156		0.07–156		0.239
Mean ± SD	30.87 ± 26.45		29.73 ± 24.86		
≤ 18	101	39.45	233	43.88	
> 18	155	60.55	298	56.12	
Gender					0.333
Female	103	40.23	233	43.88	
Male	153	59.77	298	56.12	
Clinical stage					
I	54	21.09			
II	65	25.39			
III	44	17.19			
IV	77	30.08			
4s	9	3.52			
NA	7	2.73			
Site of origin					
Adrenal gland	46	17.97			
Retroperitoneal region	87	33.98			
Mediastinum	90	35.16			
Other region	25	9.77			
NA	8	3.13			

# Two-sided *χ*^2^ test for distributions between neuroblastoma patients and controls.

### Associations between *LMO1* gene polymorphisms and neuroblastoma susceptibility

As shown in Table 2, all the observed genotype frequency distribution of the four SNPs were in accordance with Hardy-Weinberg equilibrium (HWE) in controls subjects (*P* = 0.248 for rs110419 A > G, *P* = 0.199 for rs4758051 G > A, *P* = 0.070 for rs10840002 A > G and *P* = 0.153 for rs204938 A > G). Of the four investigated SNPs, significant difference in the genotype distributions between neuroblastoma cases and controls was only observed for the rs110419 A > G polymorphism (*P* = 0.014). After adjustment for age and gender, carriers of rs110419 G allele had odds ratios (ORs) of 0.58 to 0.65 for developing neuroblastoma [AG vs. AA: adjusted OR = 0.65, 95% confidence interval (CI) = 0.47–0.91, *P* = 0.011; GG vs. AA: adjusted OR = 0.58, 95% CI = 0.36–0.91, *P* = 0.018; AG/GG vs. AA: adjusted OR = 0.63, 95% CI = 0.46–0.86, *P* = 0.004], when compared with the carriers of rs110419 AA genotype, suggesting a protective effect of this SNP against neuroblastoma. However, no association was found for the three remaining polymorphisms. While protective genotypes of the four SNPs were combined, we found the individuals with 4 protective genotypes experienced a significantly decreased neuroblastoma risk when compared with those with 0–3 protective genotypes (Adjusted OR = 0.51, 95% CI = 0.32–0.81, *P* = 0.004).

**Table 2:** Genotype and allele frequencies of the four selected polymorphisms and neuroblastoma susceptibility in a chinese population

Genotype	Cases (*N* = 256)	Controls (*N* = 531)	*P*^a^	Crude OR (95% CI)	*P*	Adjusted OR (95% CI)^b^	*P*^b^
rs110419 (HWE = 0.248)							
AA	103 (40.23)	159 (29.94)		1.00		1.00	
AG	117 (45.70)	275 (51.79)		**0.66 (0.47–0.91)**	**0.012**	**0.65 (0.47–0.91)**	**0.011**
GG	36 (14.06)	97 (18.27)		**0.57 (0.36–0.90)**	**0.017**	**0.58 (0.36–0.91)**	**0.018**
Additive			0.014	**0.73 (0.59–0.92)**	**0.006**	**0.73 (0.59–0.92)**	**0.006**
Dominant	153 (59.77)	372 (70.04)	0.004	**0.64 (0.47–0.87)**	**0.004**	**0.63 (0.46–0.86)**	**0.004**
Recessive	220 (85.94)	434 (81.73)	0.140	0.73 (0.48–1.11)	0.141	0.74 (0.49–1.12)	0.152
rs4758051 (HWE = 0.199)							
GG	95 (37.11)	194 (36.53)		1.00		1.00	
AG	126 (49.22)	242 (45.57)		1.06 (0.77–1.47)	0.713	1.08 (0.78–1.50)	0.654
AA	35 (13.67)	95 (17.89)		0.75 (0.48–1.19)	0.224	0.76 (0.48–1.21)	0.247
Additive			0.306	0.91 (0.73–1.12)	0.369	0.91 (0.74–1.13)	0.409
Dominant	161 (62.89)	337 (63.47)	0.876	0.98 (0.72–1.33)	0.875	0.99 (0.73–1.35)	0.942
Recessive	221 (86.33)	436 (82.11)	0.135	0.73 (0.48–1.11)	0.137	0.73 (0.48–1.11)	0.144
rs10840002 (HWE = 0.070)							
AA	90 (35.16)	182 (34.27)		1.00		1.00	
AG	124 (48.44)	240 (45.20)		1.05 (0.75–1.46)	0.796	1.06 (0.76–1.48)	0.741
GG	42 (16.41)	109 (20.53)		0.78 (0.50–1.21)	0.263	0.79 (0.51–1.22)	0.281
Additive			0.375	0.91 (0.74–1.12)	0.359	0.91 (0.74–1.12)	0.388
Dominant	166 (64.84)	349 (65.73)	0.808	0.96 (0.70–1.32)	0.807	0.97 (0.71–1.33)	0.863
Recessive	214 (83.59)	422 (79.47)	0.169	0.76 (0.51–1.13)	0.170	0.76 (0.51–1.13)	0.174
rs204938 (HWE = 0.153)							
AA	164 (64.06)	354 (66.67)		1.00		1.00	
AG	83 (32.42)	165 (31.07)		1.09 (0.79–1.50)	0.617	1.09 (0.79–1.50)	0.609
GG	9 (3.52)	12 (2.26)		1.62 (0.67–3.92)	0.285	1.59 (0.66–3.86)	0.304
Additive			0.523	1.14 (0.87–1.51)	0.343	1.14 (0.87–1.50)	0.349
Dominant	92 (35.94)	177 (33.33)	0.471	1.12 (0.82–1.53)	0.471	1.12 (0.82–1.54)	0.470
Recessive	247 (96.48)	519 (97.74)	0.306	1.58 (0.66–3.79)	0.310	1.55 (0.64–3.73)	0.330
Combined effect of protective genotypes							
0–3	230 (89.84)	435 (81.92)		1.00		1.00	
4	26 (10.16)	96 (18.08)	0.004	**0.51 (0.32–0.81)**	**0.005**	**0.51 (0.32–0.81)**	**0.004**

a *χ*^2^ test for genotype distributions between neuroblastoma patients and controls

b Adjusted for age and gender.

### Stratification analysis

We further explored the association between *LMO1* gene rs110419 A > G polymorphism and combined effects of protective genotypes with neuroblastoma susceptibility in stratification analysis by age, gender, sites of origin, and clinical stages (Table 3). Compared to the rs110419 AA genotype, the protective effect of AG/GG genotypes was more predominant for children > 18 months of age (adjusted OR = 0.54, 95% CI = 0.36–0.80, *P* = 0.003) and males (adjusted OR = 0.63, 95% CI = 0.42–0.95, *P* = 0.026). In term of sites of origin, we observed a significantly decreased risk of tumor developed in adrenal gland (adjusted OR = 0.36, 95% CI = 0.19–0.66, *P* = 0.001) and mediastinum (adjusted OR = 0.59, 95% CI = 0.37–0.93, *P* = 0.024), but no alteration in the risk of tumor in retroperitoneal and other regions. Moreover, we observed the AG/GG genotypes carriers had a significantly decreased risk of clinical stages III/IV neuroblastoma (adjusted OR = 0.56, 95% CI = 0.37–0.84, *P* = 0.005) when compared with the AA genotype carriers. In addition, combined analysis indicated that the 4 protective genotypes collectively decreased neuroblastoma risk in the children > 18 months of age (adjusted OR = 0.45, 95% CI = 0.24–0.86, *P* = 0.015), males (adjusted OR = 0.42, 95% CI = 0.23–0.76, *P* = 0.004), patients with tumor in mediastinum (adjusted OR = 0.27, 95% CI = 0.11–0.68, *P* = 0.006), and subgroup with early clinical stages (adjusted OR = 0.42, 95% CI = 0.22–0.82, *P* = 0.010).

**Table 3:** Stratification Analysis Of Risk Genotypes With Neuroblastoma Susceptibility

Variables	rs110419(cases/controls)		OR	*P*	Adjusted OR a	*P*^a^	Combined		OR	*P*	Adjusted OR^a^	*P*^a^
	AA	AG/GG	(95% CI)		(95% CI)		0–3	4	(95% CI)		(95% CI)	
Age, month												
≤ 18	37/74	64/159	0.81 (0.49–1.31)	0.386	0.80 (0.49–1.31)	0.378	88/187	13/46	0.60 (0.31–1.17)	0.134	0.59 (0.30–1.16)	0.124
> 18	66/85	89/213	**0.54 (0.36–0.81)**	**0.003**	**0.54 (0.36–0.80)**	**0.003**	142/248	13/50	**0.45 (0.24–0.87)**	**0.016**	**0.45 (0.24–0.86)**	**0.015**
Gender												
Females	43/73	60/160	0.64 (0.39–1.03)	0.065	0.64 (0.40–1.03)	0.068	92/199	11/34	0.70 (0.34–1.44)	0.334	0.71 (0.34–1.46)	0.346
Males	60/86	93/212	**0.63 (0.42–0.95)**	**0.027**	**0.63 (0.42–0.95)**	**0.026**	138/236	15/62	**0.41 (0.23–0.76)**	**0.004**	**0.42 (0.23–0.76)**	**0.004**
Sites of origin												
Adrenal gland	25/159	21/372	**0.36 (0.20–0.66)**	**0.001**	**0.36 (0.19–0.66)**	**0.001**	42/435	4/96	0.43 (0.15–1.23)	0.117	0.43 (0.15–1.24)	0.117
Retroperitoneal	26/159	61/372	1.00 (0.61–1.65)	0.991	1.00 (0.61–1.65)	0.993	76/435	11/96	0.66 (0.34–1.28)	0.217	0.65 (0.33–1.27)	0.203
Mediastinum	38/159	52/372	**0.59 (0.37–0.92)**	**0.022**	**0.59 (0.37–0.93)**	**0.024**	85/435	5/96	**0.27 (0.11–0.68)**	**0.005**	**0.27 (0.11–0.68)**	**0.006**
Others	12/159	13/372	0.46 (0.21–1.04)	0.061	0.46 (0.20–1.03)	0.058	23/435	2/96	0.39 (0.09–1.70)	0.212	0.37 (0.09–1.61)	0.185
Clinical stage												
I + II + 4s	46/159	80/372	0.74 (0.50–1.12)	0.154	0.74 (0.49–1.11)	0.140	115/435	11/96	**0.43 (0.23–0.84)**	**0.013**	**0.42 (0.22–0.82)**	**0.010**
III + IV	52/159	69/372	**0.57 (0.38–0.85)**	**0.006**	**0.56 (0.37–0.84)**	**0.005**	107/435	14/96	0.59 (0.33–1.08)	0.087	0.61 (0.33–1.12)	0.108

a Adjusted for age and gender.

The false-positive report probability (FPRP) values for the notable findings at different prior probability levels were shown in Table 4. Overall, FPRP analysis indicated that at the prior probability level of 0.1, significance of most of the statistically significant findings disappeared except for the decreased risk observed for carriers of rs110419 AG genotype (FPRP = 0.169) and AG/GG genotypes (FPRP = 0.093) when compared to carriers of the AA genotype. As to the stratification analyses, we found that only the association between the AG/GG genotypes and the decreased neuroblastoma risk in children of age > 18 months was still noteworthy (FPRP = 0.143). Most of the significant findings being not noteworthy in FPRP analysis may be ascribed to the limited sample sizes in the current study, especially in the subgroup. Therefore, the significant findings derived from the current study need further validation in prospective studies with large sample size.

**Table 4:** False-positive report probability values for the associations between neuroblastoma susceptibility and the frequency of genotypes of the *LMO1* gene

Genotype	Crude OR (95% CI)	*P*^a^	Statistical power^b^	Prior probability				
				0.25	0.1	0.01	0.001	0.0001
*LMO1* rs110419 A > G								
AG vs. AA	0.66 (0.47–0.91)	0.012	0.544	**0.064**	**0.169**	0.691	0.958	0.996
GG vs. AA	0.57 (0.36–0.90)	0.017	0.284	**0.149**	0.344	0.852	0.983	0.998
AG/GG vs. AA	0.64 (0.47–0.87)	0.004	0.368	**0.033**	**0.093**	0.531	0.919	0.991
AG/GG vs. AA								
> 18	0.54 (0.36–0.81)	0.003	0.140	**0.053**	**0.143**	0.647	0.949	0.995
Males	0.63 (0.42–0.95)	0.027	0.382	**0.173**	0.386	0.873	0.986	0.999
Adrenal gland	0.36 (0.20–0.66)	0.001	0.027	**0.098**	0.247	0.783	0.973	0.997
Mediastinum	0.59 (0.37–0.92)	0.022	0.285	**0.185**	0.406	0.882	0.987	0.999
Stage III + IV	0.57 (0.38–0.85)	0.006	0.215	**0.079**	0.204	0.738	0.966	0.996
*Protective genotypes*								
4 vs. 0–3	0.51 (0.32–0.81)	0.005	0.148	**0.084**	0.215	0.751	0.968	0.997
> 18	0.45 (0.24–0.87)	0.016	0.141	0.258	0.510	0.92	0.991	0.999
Males	0.41 (0.23–0.76)	0.004	0.076	**0.139**	0.326	0.842	0.982	0.998
Mediastinum	0.27 (0.11–0.68)	0.005	0.040	0.284	0.543	0.929	0.992	0.999
Stage I + II + 4s	0.43 (0.23–0.84)	0.013	0.115	0.248	0.497	0.916	0.991	0.999

a Chi-square test was used to calculate the genotype frequency distributions

b Statistical power was calculated using the number of observations in the subgroup and the OR and *P* values in this table.

## DISCUSSION

In the current case-control study with 256 neuroblastoma cases and 531 healthy controls, we verified that the *LMO1* rs110419 A > G polymorphism was associated with a decreased neuroblastoma risk. To the best of our knowledge, this is the first study to investigate the association between *LMO1* gene polymorphisms and neuroblastoma susceptibility in Southern Chinese children.

*LMO1* gene is located on 11p15, which encodes a cysteine-rich transcriptional regulator comprising two LIM zinc-binding domains. LMO family has another three superfamily numbers, LMO2, LMO3 and LMO4 [26, 27]. The LMO1 is mainly expressed in the nervous system, and involved in the nervous system development [28]. Null mutation of the *LMO4* gene, or the *LMO1*/*LMO3* genes could lead to perinatal lethality in mice [29], while the homozygous *LMO1* gene mutant mice show no overt phenotype [29]. *LMO1* gene can act together with *SCL* oncogene to facilitate the expansion of primitive thymocyte progenitors and interrupt later stages of differentiation [30]. Common genetic variants in the *LMO1* gene may increase the risk of relevant diseases through a cis-acting effect on the regulation of expression or function of LMO1 [21]. Aberrant *LMO1* locus resulting from a duplication event was associated with more advanced disease and unfavorable survival in neuroblastoma patients [21]. Apart from neuroblastoma, the *LMO1* gene polymorphisms were also associated with acute lymphoblastic leukemia susceptibility [31]. Additionally, recent studies indicated that in the anti-EGFR therapy, overexpression of LMO1 may be a predictive marker for the colorectal cancer [32], lung cancer [33], and prostate cancer [34].

GWAS is a hypothesis-free and powerful method to discovery inherited genetic variations that are associated with human disease susceptibility [19]. In the first GWAS carried out in European descent with a total of 1752 neuroblastoma cases and 4171 controls, three SNPs in the *CASC15* gene were found to be associated with increased neuroblastoma risk [1]. The association was verified by the replication studies conducted in Italians [23] and Chinese children [18, 24], but not in African-Americans [22]. In the extended GWAS by Wang et al. [21], a total of 2251 neuroblastoma patients and 6097 controls of European ancestry were enrolled. They further found that the *LMO1* gene polymorphisms were associated with neuroblastoma susceptibility, and the most significant SNP is rs110419 A > G polymorphism with a combined *P* = 5.2*10^−16^. The association between *LMO1* gene polymorphisms and neuroblastoma susceptibility was confirmed by their following expanded GWAS study with a total of 2817 neuroblastoma cases and 7473 controls [35].

In the validation study in African-Americans with a total of 390 cases and 2500 controls, Latorre et al. [22] failed to replicate any association between the four SNPs in the *LMO1* gene and neuroblastoma susceptibility. In another case-control study with 370 cases and 809 controls from Italy, Capasso et al. [23] chose two most significant SNPs (rs110419 A > G and rs4758051 G > A) to assess the association with neuroblastoma susceptibility. They found that the rs110419 A > G polymorphism, but not rs4758051 G > A, was associated with neuroblastoma susceptibility. In the study among Northern Chinese subjects, Lu et al. [24] genotyped 26 SNPs in a total of 244 neuroblastoma cases and 305 controls, including the four SNPs (rs110419 A > G, rs4758051 G > A, rs10840002 A > G and rs204938 A > G) discovered by the previous GWAS study. Totally, 11 out of 26 SNPs showed association with neuroblastoma susceptibility. They observed the significant association with rs110419 A > G and rs204938 A > G, but not with other two GWAS-identified polymorphisms (rs4758051 G > A and rs10840002 A > G). In our study conducted in Southern Chinese children, we was only able to validate the association for the most noteworthy SNP, rs110419 A > G, but failed to repeat the association between the rest three polymorphism and neuroblastoma risk. Failure to replicate the association with the SNPs in previous studies as well as ours may be ascribed to the relative weak effect of the GWAS-identified SNPs (*P* = 5.2*10^−16^ for rs110419 A > G, while *P* = 1.4*10^−11^ for rs4758051 G > A, *P* = 1.7*10^−7^ for rs204938 A > G, and *P* = 8.5*10^−7^ for rs10840002 A > G polymorphism), ethnicity difference (the previous finding were from European descent), and limited sample sizes in most of the validation studies. In the FPRP analysis, most of the significant findings in the current study appeared to be not noteworthy at the FPRP threshold of 0.2, which may be due to the limited sample size in each stratum. Aberrant *LMO1* was associated with more advanced disease [21], and the ancestral rs2168101 G allele was associated with tumor formation [36]. We found that the rs110419 AG/GG genotypes were associated with decreased neuroblastoma susceptibility in patients with advanced stage neuroblastoma. However, when we performed FPRP analysis, the association appeared to be not noteworthy at the FPRP threshold of 0.2. Therefore, the conclusions drawn from the current study should be interpreted cautiously. In the future, the studies with much larger sample size are encouraged to validate our findings.

Though this study is the largest one performed in Chinese children and the first investigation in Southern Chinese subjects, certain limitations should be acknowledged. First, the sample size in the current study is still not large enough, because of the very low incidence rate of neuroblastoma. Therefore, multicenter studies with larger sample size are needed to confirm the roles of *LMO1* in neuroblastoma susceptibility. Second, we only tested the four *LMO1* SNPs that were discovered by previous GWAS. None of these SNPs is potentially functional. More potentially functional SNPs located in the *LMO1* gene should be investigated, such as the rs2168101 G > T polymorphism that was found to be associated with neuroblastoma recently [36]. Finally, in the current study, we only adjusted for age and gender in the logistic regression analysis. Due to the nature of retrospective study, we were not able to collect and control for other factors, such as the dietary intakes as well as the environment exposure for their parents and the children.

In summary, the present hospital-based case-control study confirmed that the *LMO1* gene rs110419 G allele was associated with decreased neuroblastoma susceptibility in a Southern Chinese population. However, future studies with larger sample size and functional experiments should be conducted to further explore the role of *LMO1* and underlying mechanisms in neuroblastoma carcinogenesis.

## MATERIALS AND METHODS

### Study population

In the current study, all the neuroblastoma cases and healthy controls were restricted to unrelated ethnic Chinese Han. A total of 256 patients with neuroblastoma were mainly recruited at the Department of Pediatric Surgery of the Guangzhou Women and Children's Medical Center between February 2010 and November 2015, as we described previously [18, 37]. All of the neuroblastoma cases were newly diagnosed and histopathologically confirmed, and had not previous history of other cancers. No restriction was applied regarding age, gender, or disease stage at the time of recruiting neuroblastoma cases. The 531 age-, gender-, and ethnicity-matched healthy controls were also collected from the Guangzhou Women and Children's Medical Center as described elsewhere [18, 37]. All the included subjects provided written informed consent signed by their parents or guardians. This study was approved by the Institutional Review Board of Guangzhou Women and Children's Medical Center (GZR2015-099).

### SNP selection and genotyping

We chose all of the four SNPs (rs110419 A > G, rs4758051 G > A, rs10840002 A > G and rs204938 A > G) in the *LMO1* gene identified by a previous GWAS study [21]. These four SNPs can also capture an additional of 10 SNPs with a linkage disequilibrium (LD) > 0.6 (Supplementary Table 1). We performed Taqman real-time PCR assay to genotype these SNPs as we described previously [18, 38]. Briefly, high-quality DNA samples were genotyped using Taqman real-time PCR method on a 7900 HT sequence detector system (Applied Biosystems, Foster City, CA, USA). Eight positive controls and eight negative controls were included in each 384-well plate. Additionally, 10% samples were randomly selected and repeated, and the reproducibility was 100% concordant.

### Statistical analysis

Genotype frequencies of each SNP as well as the demographic variables (e.g., age and gender) between neuroblastoma cases and healthy controls were compared using the χ^2^ test. ORs and corresponding 95% CIs were calculated by unconditional logistic regression analyses adjusted for age and gender. Genotypic frequencies in controls for each SNP were tested for departure from HWE using goodness-of-fit χ^2^ test. The FPRP was calculated for all significant findings as described previously [39–41]. We preset 0.2 as a FPRP threshold and chose a prior probability of 0.1 to detect OR of 0.67 (for protective effects). Association with FPRP value less than 0.2 was recognized as noteworthy. Statistical analyses were performed using SAS software (Version 9.1; SAS Institute, Cary, NC). All *P* values in the current study were two-sided, and a *P* value of less than 0.05 was considered as statistical significance.

## SUPPLEMENTARY MATERIALS TABLE


