# Supplementary material for: LMO1 gene polymorphisms contribute to decreased neuroblastoma susceptibility in a Southern Chinese population
Source: Oncotarget. 2016 Mar 18;7(16):22770–8. doi: 10.18632/oncotarget.8178 (PMC5008399; doi:10.18632/oncotarget.8178)
Supplement: Supplementary file 1 [file oncotarget-07-22770-s001.pdf]

LMO1 gene polymorphisms contribute to decreased neuroblastoma susceptibility in a Southern Chinese population

Supplementary Materials

Supplementary Table S1: SNPs captured by the four GWAS identified SNPs as predicted by SNPinfo (http://snpinfo.niehs.nih.gov/) software

<table>
<tr><th>rs[a]</th><th>Chr</th><th>Allele</th><th>LDsnp</th><th>Pop/LD</th><th>TFBS</th><th>Splicing (ESE or ESS)</th><th>miRNA (miRanda)</th><th>nsSNP</th><th>Reg Potential</th><th>Conservation</th><th>Nearby Gene</th><th>Distance (bp)</th><th>Allele</th><th>Asian</th><th>CHB</th></tr>
<tr><td>rs10840002</td><td>11</td><td>A/G</td><td>rs10840002|rs4758051</td><td>1|CHB/0.825</td><td>—</td><td>—</td><td>—</td><td>—</td><td>0.103058</td><td>0</td><td>RIC3||LMO1</td><td>−52454||−2831</td><td>A</td><td>0.5</td><td>0.47</td></tr>
<tr><td>rs110419</td><td>11</td><td>A/G</td><td>rs110419</td><td>1</td><td>—</td><td>—</td><td>—</td><td>—</td><td>0</td><td>0</td><td>LMO1</td><td>6996||32553</td><td>A</td><td>0.634</td><td>0.53</td></tr>
<tr><td>rs110420</td><td>11</td><td>C/T</td><td>rs110419</td><td>CHB/1.000</td><td>—</td><td>—</td><td>—</td><td>—</td><td>0</td><td>0</td><td>LMO1</td><td>7192||32357</td><td>T</td><td>0.491</td><td>0.545</td></tr>
<tr><td>rs204926</td><td>11</td><td>G/A</td><td>rs110419</td><td>CHB/0.607</td><td>—</td><td>—</td><td>—</td><td>—</td><td>0</td><td>0</td><td>LMO1</td><td>9249||30300</td><td>G</td><td>0.64</td><td>0.628</td></tr>
<tr><td>rs204928</td><td>11</td><td>G/A</td><td>rs110419</td><td>CHB/0.906</td><td>—</td><td>—</td><td>—</td><td>—</td><td>0</td><td>0.001</td><td>LMO1</td><td>8576||30973</td><td>A</td><td>0.614</td><td>0.568</td></tr>
<tr><td>rs3750952</td><td>11</td><td>G/C</td><td>rs110419</td><td>CHB/0.780</td><td>—</td><td>Y</td><td>—</td><td>—</td><td>0.524312</td><td>1</td><td>LMO1</td><td>6064||33485</td><td>G</td><td>0.632</td><td>0.614</td></tr>
<tr><td>rs4758317</td><td>11</td><td>A/C</td><td>rs110419</td><td>CHB/0.976</td><td>—</td><td>—</td><td>—</td><td>—</td><td>0</td><td>0</td><td>LMO1</td><td>4954||34595</td><td>A</td><td>0.394</td><td>0.464</td></tr>
<tr><td>rs204937</td><td>11</td><td>T/C</td><td>rs204938</td><td>CHB/0.804</td><td>—</td><td>—</td><td>—</td><td>—</td><td>0.232693</td><td>0</td><td>LMO1</td><td>35081||4468</td><td>T</td><td>0.844</td><td>0.851</td></tr>
<tr><td>rs204938</td><td>11</td><td>T/C</td><td>rs204938</td><td>1</td><td>—</td><td>—</td><td>—</td><td>—</td><td>0.278185</td><td>0</td><td>LMO1</td><td>32340||7209</td><td>C</td><td>0.211</td><td>0.179</td></tr>
<tr><td>rs2061037</td><td>11</td><td>A/G</td><td>rs204938</td><td>CHB/0.676</td><td>—</td><td>—</td><td>—</td><td>—</td><td>0</td><td>0</td><td>LMO1</td><td>20772||18777</td><td>G</td><td>0.833</td><td>0.827</td></tr>
<tr><td>rs484161</td><td>11</td><td>C/T</td><td>rs204938</td><td>CHB/0.648</td><td>—</td><td>—</td><td>—</td><td>—</td><td>0.373993</td><td>0</td><td>LMO1</td><td>18668||20881</td><td>T</td><td>0.18</td><td>0.202</td></tr>
<tr><td>rs10840000</td><td>11</td><td>C/G</td><td>rs4758051|rs10840002</td><td>CHB/0.950|CHB/0.756</td><td>—</td><td>—</td><td>—</td><td>—</td><td>0.106824</td><td>0</td><td>RIC3||LMO1</td><td>−49541||−5744</td><td>C</td><td>0.506</td><td>0.571</td></tr>
<tr><td>rs4758050</td><td>11</td><td>C/G</td><td>rs4758051|rs10840002</td><td>CHB/1.000|CHB/0.829</td><td>—</td><td>—</td><td>—</td><td>—</td><td>0.067678</td><td>0</td><td>RIC3||LMO1</td><td>−47973||−7312</td><td>G</td><td>0.534</td><td>0.478</td></tr>
<tr><td>rs4758051</td><td>11</td><td>A/G</td><td>rs4758051|rs10840002</td><td>1|CHB/0.825</td><td>—</td><td>—</td><td>—</td><td>—</td><td>0</td><td>0</td><td>RIC3||LMO1</td><td>−48067||−7218</td><td>G</td><td>0.528</td><td>0.518</td></tr>
</table>

SNP, single nucleotide polymorphism; GWAS, genome-wide association study; LD, linkage disequilibrium; TFBS, transcription factor binding sites; ESE, exon *splicing* enhancer; ESS, exon *splicing* silencer; CHB, Han Chinese in Beijing, China

[a]The rs in bold were discovered by previous genome-wide association study.
